# Supplementary material for: Neuroprotective gap-junction-mediated bystander transformations in the adult zebrafish spinal cord after injury
Source: Nat Commun. 2024 May 21;15:4331. doi: 10.1038/s41467-024-48729-9 (PMC11109231; doi:10.1038/s41467-024-48729-9)
Supplement: Supplementary file 1 — Supplementary Information [file 41467_2024_48729_MOESM1_ESM.pdf]

Supplementary Information:

Neuroprotective gap-junction-mediated bystander transformations in the adult zebrafish spinal cord after injury

Andrea Pedroni, Yu-Wen E. Dai, Leslie Lafouasse, Weipang Chang, Ipsit Srivastava, Lisa Del Vecchio and Konstantinos Ampatzis

Supplementary Figures: 13
Supplementary Tables: 2

Supplementary figures:

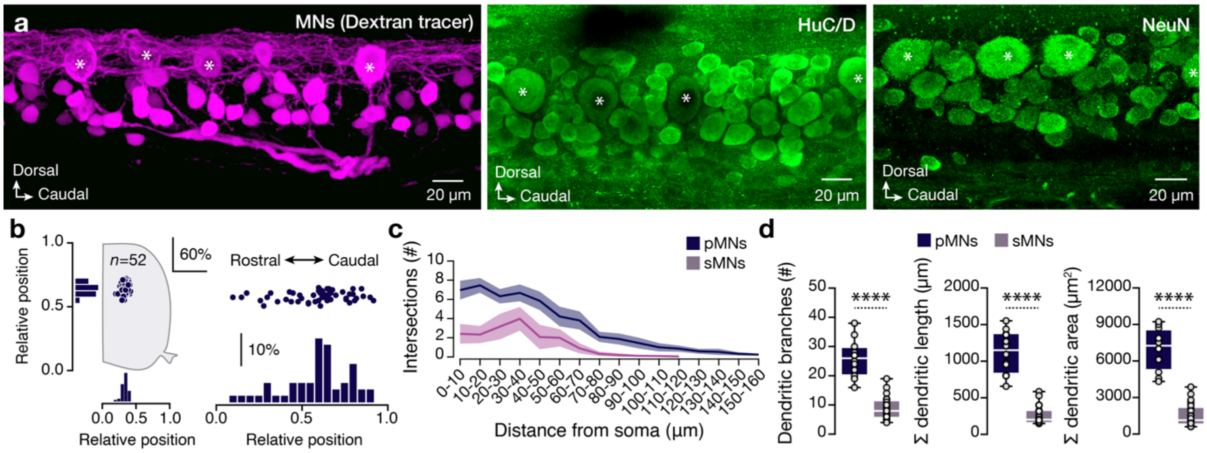

Supplementary Fig. 1. The pMNs are the largest neurons in the spinal cord with extended dendritic morphology. a. Whole mount confocal images showing the size of the pMNs in relation to other MNs or other spinal cord neurons (HuC/D+ and NeuN+). **b.** Primary MNs (pMNs) are located in the dorsal and medial part of the spinal cord hemisegment and distributed along the rostrocaudal axis of the hemisegment. **c.** Sholl analysis of the pMN and sMN dendrite intersection in relation to the distance from the soma. **d.** Analysis of the dendritic parameters between the adult zebrafish spinal primary and secondary motoneurons. Asterisks indicate the pMN cell bodies. Σ, summation (total); HuC/D, elav3+4; NeuN, neuronal protein (Fox-3/Rbfox3); pMN, primary motoneuron; sMN, secondary motoneuron. Data are presented as box plots showing the median with 25/75 percentile (box and line) and minimum–maximum (whiskers). ****$P < 0.0001$. For detailed statistics, see Supplementary Table 1. Source data are provided as a Source Data file.

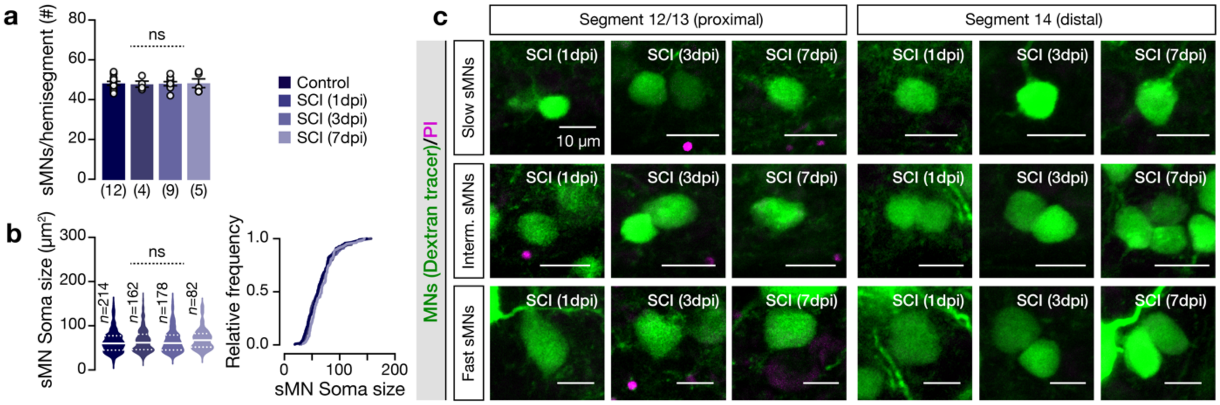

Supplementary Fig. 2. Secondary MNs (sMNs) are resilient to injury. a. Quantification of the number of the traced (Dextran retrograde tracer) secondary MNs (sMNs) in all experimental conditions. **b.** Analysis of the soma size of all retrogradely traced sMNs in uninjured (control) and injured animals. **c.** Close-up confocal images of the different functional types of the sMNs (green) show no incorporation of PI (magenta) in proximal and distal segments to the injury site. dpi, days post-injury; PI, propidium iodide; SCI, spinal cord injury; sMN, secondary motoneuron. Data are presented as mean ± s.e.m., and as violin plots. ns, not significant. For detailed statistics, see Supplementary Table 1. Source data are provided as a Source Data file.

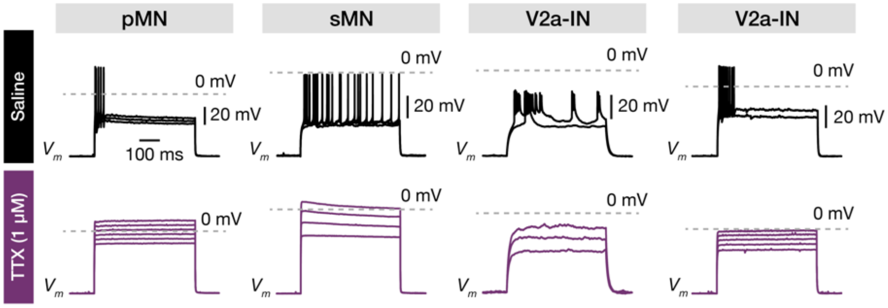

Supplementary Fig. 3. TTX eliminates spinal neuron firing. Sample current-clamp traces show that the application of Na^{+} channel antagonist tetrodotoxin (TTX) eradicated action potentials in all tested motoneuron types and the V2a-IN subpopulations following depolarizing current injections. sMN, secondary motoneuron; TTX, tetrodotoxin.

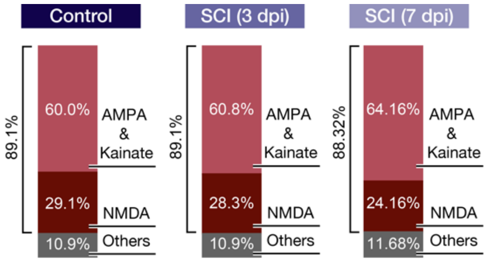

Supplementary Fig. 4. Different glutamate receptors mediate the postsynaptic events. Analysis of the proportion of the different ionotropic glutamate receptors mediating the pMN postsynaptic events in all experimental conditions, as presented in Figure 2. AMPA, α-amino-3-hydroxy-5-methyl-4-isoxazolepropionic acid; dpi, days post-injury; NMDA, *N*-methyl-d-aspartic acid.

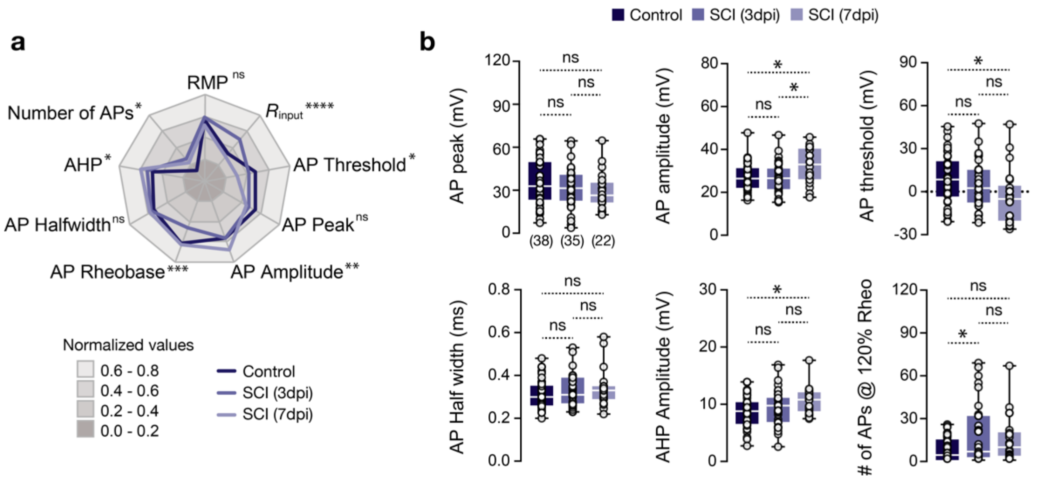

Supplementary Fig. 5. Physiological features of the adult zebrafish pMNs. a. Spider plot of the normalized mean values of pMN electrical properties in all experimental conditions. Normalizations were performed for each property to the highest obtained value. **b.** Detailed analysis of the cellular and electrical properties of the pMNs in uninjured and post-injured (3 and 7 dpi) animals. AHP, after-hyperpolarization potential; AP, action potential; dpi, days post-injury; Rheo, rheobase; *R*input, input resistance; RMP, resting membrane potential; SCI, spinal cord injury. Data are presented as box plots showing the median with 25/75 percentile (box and line) and minimum–maximum (whiskers). $^{*}P < 0.05$; $^{**}P < 0.01$; $^{***}P < 0.001$; $^{****}P < 0.0001$; ns, not significant. For detailed statistics, see Supplementary Table 1. Source data are provided as a Source Data file.

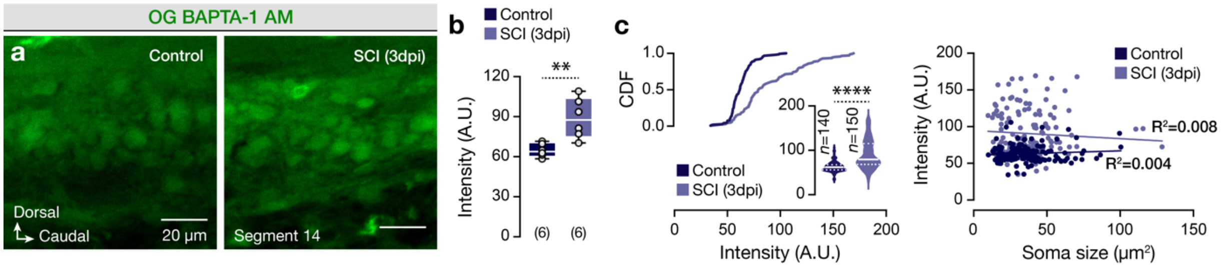

Supplementary Fig. 6. Resting calcium in adult zebrafish spinal neurons. a. Images of the adult zebrafish spinal cord after loading the cell-permeant calcium-sensitive dye Oregon green 488 BAPTA-1 in uninjured (control) and injured (3 dpi) adult zebrafish. **b.** Quantification of the resting calcium signal intensity. **c.** Quantification of the resting calcium in all individual spinal neurons and analysis of the resting calcium intensity in relation to their soma size in both experimental conditions. A.U., arbitrary unit; CDF, cumulative distribution (frequency); SCI, spinal cord injury. Data are presented as violin plots and box plots showing the median with 25/75 percentile (box and line) and minimum–maximum (whiskers). $^{**}P < 0.01$; $^{****}P < 0.0001$. For detailed statistics, see Supplementary Table 1. Source data are provided as a Source Data file.

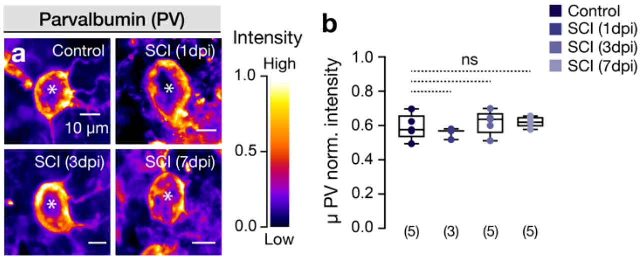

Supplementary Fig. 7. Parvalbumin expression levels remain unaltered in pMNs after injury. a. Pseudo-colored images showing the normalized intensity of immunolabelled pMNs with calcium-binding proteins PV. **b.** Quantification of the mean normalized intensity of PV in each animal for all experimental conditions. Normalizations were performed for PV calcium-binding protein to the highest obtained intensity value. Asterisks indicate the pMN cell bodies. μ, population mean; dpi, days post-injury; PV, parvalbumin; SCI, spinal cord injury. Data are presented as box plots showing the median with 25/75 percentile (box and line) and minimum–maximum (whiskers). ns, not significant. For detailed statistics, see Supplementary Table 1. Source data are provided as a Source Data file.

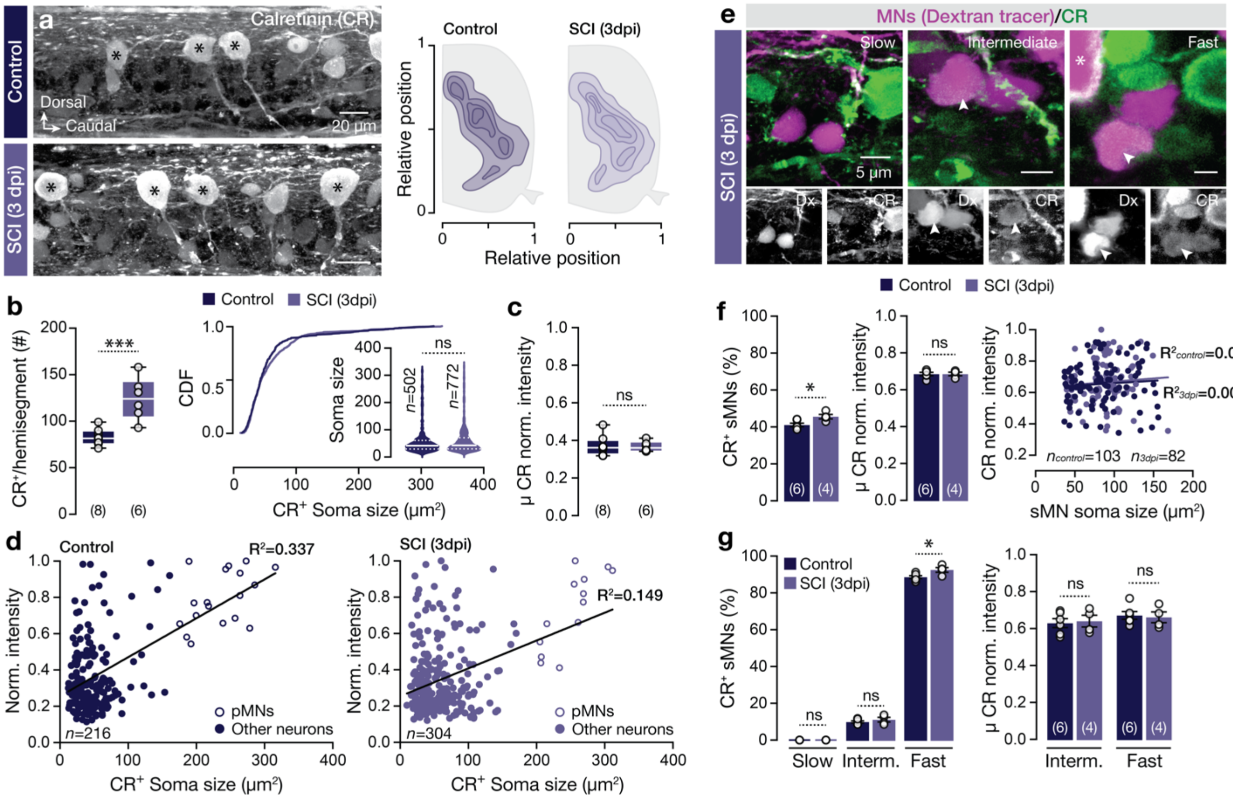

Supplementary Fig. 8. Increase of CR-positive neurons after injury. a. Confocal images from whole-mount adult zebrafish spinal cord showing the CR+ neurons in uninjured and injured (3 dpi) animals. Spatial distribution of the CR+ neurons in adult zebrafish spinal hemisegment in each condition. **b.** Quantification of the CR-expressing neurons in adult spinal cord hemisegment and their soma size. **c.** Average normalized intensity of CR+ neurons in the whole spinal cord hemisegment. **d.** The normalized CR intensity in relation to neuron soma size in uninjured and 3 dpi animals indicates that pMNs are high CR-expressing spinal neurons. **e.** Representative images show the CR expression in retrogradely labeled secondary MNs (sMNs) at 3dpi. Arrowheads indicate the double-labeled neurons. **f.** The proportion of CR+ sMNs and the mean (µ) normalized CR intensity in the whole sMN population before and after injury (3dpi). Analysis of the normalized CR intensity against the sMN soma size. **g.** Quantification (proportion) of the secondary motoneuron types (Slow, intermediate, and fast) that express CR in control and 3dpi. Analysis of the mean normalized CR intensity in intermediate and fast MN pool before and after injury (3dpi). Asterisks indicate the pMN cell bodies. µ, population mean; CDF, cumulative distribution (frequency); CR, calretinin; dpi, days post-injury; SCI, spinal cord injury. Data are presented as violin plots and as box plots showing the median with 25/75 percentile (box and line) and minimum–maximum (whiskers). **P* < 0.05; ****P* < 0.001; ns, not significant. For detailed statistics, see Supplementary Table 1. Source data are provided as a Source Data file.

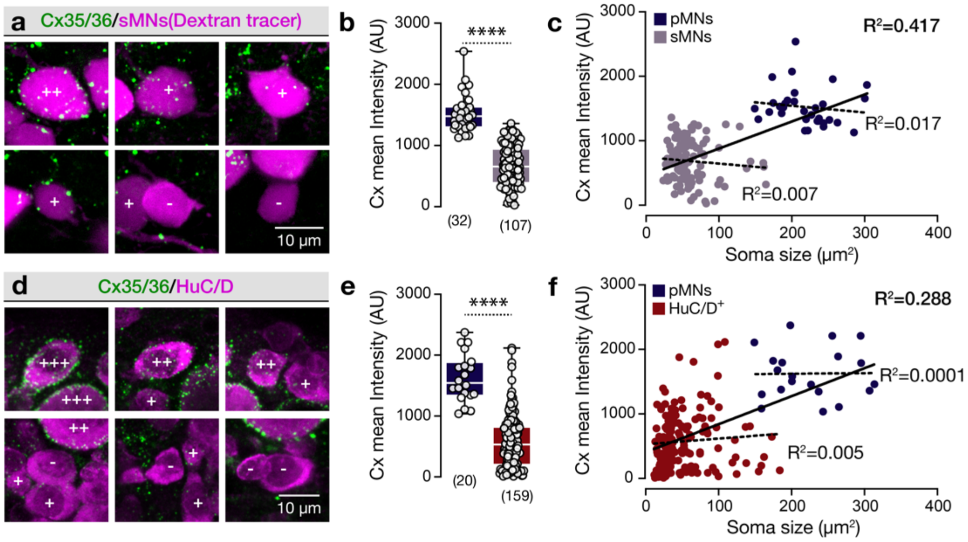

Supplementary Fig. 9. Connexin 35/36 expression in adult zebrafish spinal cord neurons. a. Cx35/36 expression (green) in spinal secondary motoneurons (sMNs; magenta). **b-c.** Cx35/36 intensity in different motoneurons (primary and secondary) and relation of the Cx35/36 intensity to motoneuron soma size. **d.** Microphotographs show the expression of Cx35/36 (green) in spinal cord neurons (HuC/D+; magenta). **e.** Quantification of the Cx35/36 intensity between the pMNs and other spinal neurons. **f.** Intensity analysis in relation to soma size between the pMNs and spinal cord neurons. Asterisks indicate the pMN cell bodies. The plus symbol indicates positive expression in the soma (+++, high; ++, medium; +, low), whereas the minus symbol (-) indicates no expression in the neuronal soma. AU, arbitrary unit; Cx, connexin; dpi, days post-injury; pMN, primary motoneuron; SCI, spinal cord injury. Data are presented as box plots showing the median with 25/75 percentile (box and line) and minimum–maximum (whiskers). ****$P < 0.0001$. For detailed statistics, see Supplementary Table 1. Source data are provided as a Source Data file.

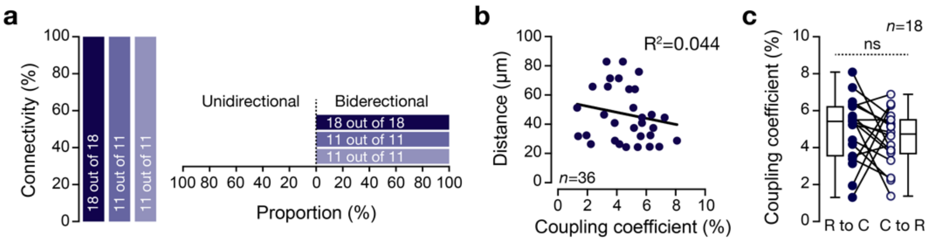

Supplementary Fig. 10. Electrical coupling between pMNs. a. Electrical coupling always exists between pMNs of the same segment, which is bidirectional. **b.** No correlation exists between the coupling coefficient and the relative distance between the recorded pMNs. **c.** Electrical coupling (coupling coefficient) remains the same regardless of the relative rostrocaudal position of the pMNs in the spinal cord. Data are presented as mean ± s.e.m. and as box plots showing the median with 25/75 percentile (box and line) and minimum–maximum (whiskers). ns, not significant. For detailed statistics, see Supplementary Table 1. Source data are provided as a Source Data file.

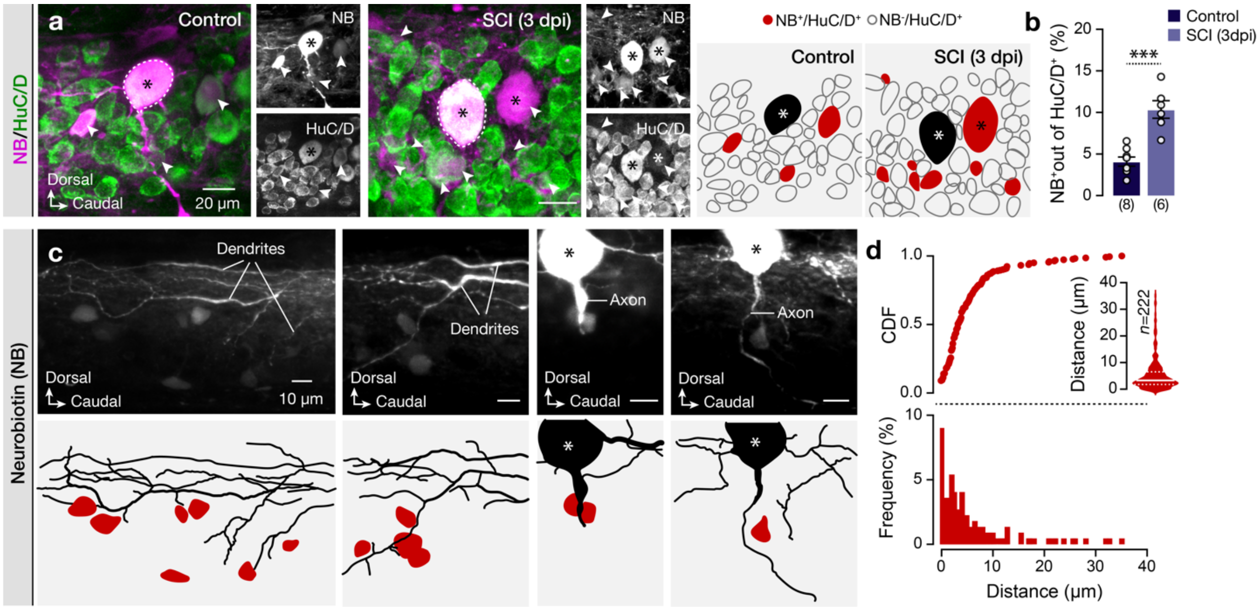

Supplementary Fig. 11. Selectivity of dye coupling from a NB-filled adult spinal pMN. a. Whole mount representative images (sample stack) and drawings showing the dye-coupled neurons ($NB^{+}HuC/D^{+}$; red filled) distribution in relation to non-dye coupled neurons ($NB^{-}HuC/D^{+}$; gray outline) from a single NB-filled pMN (in black). Arrowheads indicate the dye-coupled neurons. **b.** Quantification of the proportion of the dye-coupled neurons ($NB^{+}HuC/D^{+}$) to non-dye dye-coupled neurons ($NB^{-}HuC/D^{+}$). **c.** Confocal whole-mount images and corresponding drawings showing the proximity (distance in µm) between the NB-filled pMN structures (in black) and the somata of the dye-coupled neurons (in red). **d.** Quantification of the Euclidean distance between the dye-coupled neurons and the pMN. Asterisks indicate the pMN cell bodies. White dashed lines indicate the NB-filled pMN. dpi, days post-injury; HuC/D, elav3+4; NB, neurobiotin (N-(2-aminoethyl) biotinamide hydrochloride); SCI, spinal cord injury. Data are presented as mean ± s.e.m., and as violin plots. $^{***}P < 0.001$. For detailed statistics, see Supplementary Table 1. Source data are provided as a Source Data file.

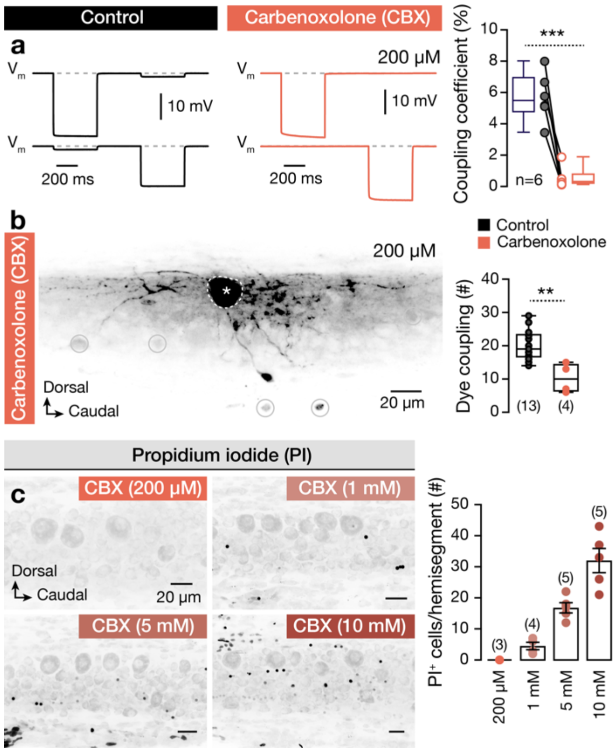

Supplementary Fig. 12. Carbenoxolone affects pMN electrical and dye coupling. a. Sample current-clamp average traces (from ~40 sweeps) showing the effectiveness of CBX as a potent blocker of the electrical coupling. Analysis of the coupling coefficient after the application of CBX. **b.** Inverted confocal image from whole mount spinal cord showing the diffusion of the NB (dye coupling, white circles) from a loaded pMN (dashed white line). Quantification of the detected dye-coupled cells. **c.** Evaluation of potential cell toxicity of CBX administration into zebrafish spinal cord. Inverted confocal images from whole-mount adult zebrafish spinal cord showing the PI^+ cells after administration of different dosages of CBX and quantification of the detected PI^+ cells per hemisegment. Asterisks indicate the pMN cell bodies. CBX, Carbenoxolone; NB, neurobiotin (N-(2-aminoethyl) biotinamide hydrochloride); PI, propidium iodide. Data are presented as mean ± s.e.m. and as box plots showing the median with 25/75 percentile (box and line) and minimum–maximum (whiskers). $^{**}P < 0.01$; $^{***}P < 0.001$. For detailed statistics, see Supplementary Table 1. Source data are provided as a Source Data file.

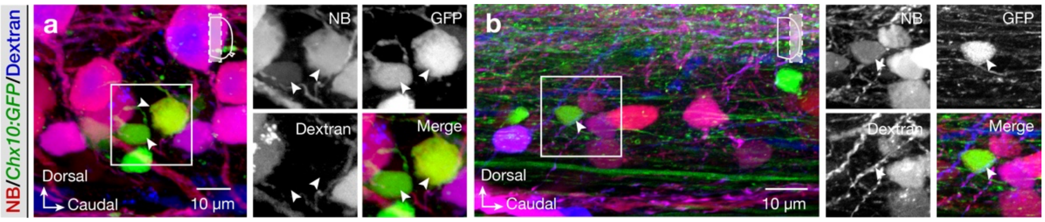

Supplementary Fig. 13. Dye-coupled neurons to motoneurons have an interneuron identity. a-b. Medial and lateral stack of whole-mount confocal images showing the retrograde traced spinal MNs (Dextran tracer; blue), the NB-filled neurons (red), and the V2a-INs (*Chx10:GFP*$^{+}$; green). Arrowheads indicate the dye-coupled V2a-INs. GFP, green fluorescent protein; NB, neurobiotin (N-(2-aminoethyl) biotinamide hydrochloride)

Supplementary Table 1. Detailed statistics

Figure	Statistics	Result	Post-hoc Test	comparison	Significance	*P-value*
**Main figures**						
**1b**	One-way ANOVA	$F_{(3, 2152)} = 2188$<br>$P < 0.0001$	Dunnett's test	pMNs<br>sMNs	****	$P_{adj} < 0.0001$
				pMNs<br>HuC/D	****	$P_{adj} < 0.0001$
				pMNs<br>NeuN	****	$P_{adj} < 0.0001$
	Descriptive	pMNs (*n* = 107 neurons): 215.0 ± 3.649<br>sMNs (*n* = 308 neurons): 57.55 ± 1.179<br>HuC/D (*n* = 1014 neurons): 43.58 ± 0.646<br>NeuN (*n* = 727 neurons): 42.13 ± 0.712				
**1e**<br>Soma size	One-way ANOVA	$F_{(7, 44)} = 0.374$			ns	$P = 0.9125$
	Descriptive	**Segment 13**<br>Control (*n* = 9 zebrafish): 212.2 ± 6.66<br>SCI-1dpi (*n* = 5 zebrafish): 218.3 ± 3.28<br>SCI-3dpi (*n* = 5 zebrafish): 215.7 ± 6.41<br>SCI-7dpi (*n* = 7 zebrafish): 221.7 ± 6.22<br>**Segment 14**<br>Control (*n* = 9 zebrafish): 211.8 ± 6.99<br>SCI-1dpi (*n* = 5 zebrafish): 216.9 ± 6.43<br>SCI-3dpi (*n* = 5 zebrafish): 217.6 ± 3.69<br>SCI-7dpi (*n* = 7 zebrafish): 221.1 ± 6.59				
**1f**	One-way ANOVA	$F_{(2, 8)} = 37.23$<br>$P < 0.0001$	Holm-Šidák's test	SCI-1dpi<br>SCI-3dpi	***	$P_{adj} = 0.0005$
				SCI-1dpi<br>SCI-7dpi	***	$P_{adj} = 0.0002$
				SCI-3dpi<br>SCI-7dpi	ns	$P_{adj} = 0.1960$
	Descriptive	SCI-1dpi (*n* = 5 zebrafish): 288.4 ± 26.1<br>SCI-3dpi (*n* = 3 zebrafish): 91 ± 7.2<br>SCI-7dpi (*n* = 3 zebrafish): 41.3 ± 11.8				
**1j**<br>Soma size	One-way ANOVA	$F_{(2, 20)} = 0.1112$			ns	$P = 0.8953$
	Descriptive	Control (*n* = 12 pMNs): 230.6 ± 9.63<br>SCI-3dpi (*n* = 6 pMNs): 238.9 ± 17.51<br>SCI-7dpi (*n* = 5 pMNs): 232.5 ± 13.64				
**1j**<br>Dendritic branches	One-way ANOVA	$F_{(2, 20)} = 1.001$			ns	$P = 0.3851$
	Descriptive	Control (*n* = 12 pMNs): 25.83 ± 1.81<br>SCI-3dpi (*n* = 6 pMNs): 29.33 ± 2.44<br>SCI-7dpi (*n* = 5 pMNs): 24 ± 3.36				
**1j**<br>Dendritic length	One-way ANOVA	$F_{(2, 20)} = 0.043$			ns	$P = 0.9573$
	Descriptive	Control (*n* = 12 pMNs): 1124 ± 82.69<br>SCI-3dpi (*n* = 6 pMNs): 1164 ± 93.55<br>SCI-7dpi (*n* = 5 pMNs): 1152 ± 149.4				
**1j**<br>Dendritic area	One-way ANOVA	$F_{(2, 20)} = 0.534$			ns	$P = 0.5943$
	Descriptive	Control (*n* = 12 pMNs): 7021± 486<br>SCI-3dpi (*n* = 6 pMNs): 7693 ± 710.6<br>SCI-7dpi (*n* = 5 pMNs): 6712 ± 642.6				
**2c**<br>Frequency	One-way ANOVA	$F_{(2, 60)} = 16.92$<br>$P < 0.0001$	Tukey's test	Control<br>SCI-3dpi	****	$P_{adj} < 0.0001$
				Control<br>SCI-7dpi	ns	$P_{adj} = 0.702$
				SCI-3dpi<br>SCI-7dpi	***	$P_{adj} = 0.0006$
	Descriptive	Control (*n* = 22 pMNs): 17.14 ± 1.18<br>SCI-3dpi (*n* = 27 pMNs): 28.91 ± 1.6<br>SCI-7dpi (*n* = 14 pMNs): 19.19 ± 2.22				
**2c**<br>Amplitude<br>Average values	One-way ANOVA	$F_{(2, 42)} = 9782$<br>$P = 0.0003$	Tukey's test	Control<br>SCI-3dpi	***	$P_{adj} = 0.0006$
				Control<br>SCI-7dpi	ns	$P_{adj} = 0.9868$

				SCI-3dpi<br>SCI-7dpi	**	P_{adj} = 0.0074
	Descriptive	Control (*n* = 17 pMNs): 0.269 ± 0.01<br>SCI-3dpi (*n* = 19 pMNs): 0.399 ± 0.02<br>SCI-7dpi (*n* = 9 pMNs): 0.275 ± 0.03				
**2c**<br>Amplitude<br>All values	One-way ANOVA	$F_{(2, 2435)}$ = 13.86<br>*P* < 0.0001	Tukey's test	Control<br>SCI-3dpi	****	P_{adj} < 0.0001
				Control<br>SCI-7dpi	ns	P_{adj} = 0.2096
				SCI-3dpi<br>SCI-7dpi	**	P_{adj} = 0.0094
	Descriptive	Control (*n* = 778 EPSPs): 0.281 ± 0.01<br>SCI-3dpi (*n* = 1074 EPSPs): 0.366 ± 0.01<br>SCI-7dpi (*n* = 586 EPSPs): 0.313 ± 0.01				
**2e**<br>Frequency	One-way ANOVA	$F_{(2, 16)}$ = 7.71<br>*P* = 0.0045	Tukey's test	Control<br>SCI-3dpi	*	P_{adj} = 0.018
				Control<br>SCI-7dpi	ns	P_{adj} = 0.748
				SCI-3dpi<br>SCI-7dpi	**	P_{adj} = 0.005
	Descriptive	Control (*n* = 7 pMNs): 17.14 ± 2.9<br>SCI-3dpi (*n* = 6 pMNs): 32.72 ± 4.7<br>SCI-7dpi (*n* = 6 pMNs): 13.44 ± 2.9				
**2e**<br>Amplitude<br>Average values	One-way ANOVA	$F_{(2, 16)}$ = 6.013<br>*P* = 0.0113	Tukey's test	Control<br>SCI-3dpi	*	P_{adj} = 0.0213
				Control<br>SCI-7dpi	ns	P_{adj} = 0.9900
				SCI-3dpi<br>SCI-7dpi	*	P_{adj} = 0.0205
	Descriptive	Control (*n* = 7 pMNs): 13.02 ± 1.43<br>SCI-3dpi (*n* = 6 pMNs): 20.59 ± 2.35<br>SCI-7dpi (*n* = 6 pMNs): 12.68 ± 1.57				
**2e**<br>Amplitude<br>All values	One-way ANOVA	$F_{(2, 890)}$ = 17.72<br>*P* < 0.0001	Tukey's test	Control<br>SCI-3dpi	*	P_{adj} = 0.0489
				Control<br>SCI-7dpi	**	P_{adj} = 0.0026
				SCI-3dpi<br>SCI-7dpi	****	P_{adj} < 0.0001
	Descriptive	Control (*n* = 261 EPSCs): 18.52 ± 0.87<br>SCI-3dpi (*n* = 382 EPSCs): 21.51 ± 0.97<br>SCI-7dpi (*n* = 250 EPSCs): 13.87 ± 0.71				
**2g**<br>Control frequency	Paired *t*-test	t = 1.562, df = 5 (Two-tailed)		Saline<br>TTX	ns	*P* = 0.1791
	Descriptive	Saline (*n* = 6 pMNs): 18.39 ± 3.11<br>TTX (*n* = 6 pMNs): 14.19 ± 2.6<br>TTX – Saline (*n* = 6 pMNs): -4.2 ± 2.68				
**2g**<br>Control amplitude	Paired *t*-test	t = 0.702, df = 5 (Two-tailed)		Saline<br>TTX	ns	*P* = 0.5136
	Descriptive	Saline (*n* = 6 pMNs): 12.66 ± 1.47<br>TTX (*n* = 6 pMNs): 11.48 ± 0.5<br>TTX – Saline (*n* = 6 pMNs): -1.17 ± 1.67				
**2g**<br>3dpi frequency	Paired *t*-test	t = 0.979, df = 6 (Two-tailed)		Saline<br>TTX	ns	*P* = 0.3651
	Descriptive	Saline (*n* = 7 pMNs): 33.43 ± 3.87<br>TTX (*n* = 7 pMNs): 32.53 ± 3.78<br>TTX – Saline (*n* = 7 pMNs): -0.9 ± 0.92				
**2g**<br>3dpi amplitude	Paired *t*-test	t = 2.365, df = 6 (Two-tailed)		Saline<br>TTX	ns	*P* = 0.0559
	Descriptive	Saline (*n* = 7 pMNs): 20.85 ± 2.04<br>TTX (*n* = 7 pMNs): 16.85 ± 1.71<br>TTX – Saline (*n* = 7 pMNs): -4.0 ± 1.69				
**2i**<br>Control<br>Frequency	RM<br>One-way<br>ANOVA	$F_{(1.42, 7.12)}$ = 36.9,<br>*P* = 0.0003	Tukey's test	TTX<br>TTX+NBQX	**	P_{adj} = 0.007
				TTX<br>TTX+NBQX+AP5	**	P_{adj} = 0.0023
				TTX+NBQX<br>TTX+NBQX+AP5	*	P_{adj} = 0.0164
	Descriptive	TTX (*n* = 6 pMNs): 16.38 ± 2.3<br>TTX+NBQX (*n* = 6 pMNs): 6.58 ± 1.32<br>TTX+NBQX+AP5 (*n* = 6 pMNs): 1.79 ± 0.76				

**2i**<br>SCI-3dpi<br>Frequency	RM<br>One-way<br>ANOVA	$F_{(1.49,\ 8.98)} = 92.47$,<br>$P < 0.0001$	Tukey's test	TTX<br>TTX+NBQX	***	$P_{adj} = 0.0002$
				TTX<br>TTX+NBQX+AP5	****	$P_{adj} < 0.0001$
				TTX+NBQX<br>TTX+NBQX+AP5	**	$P_{adj} = 0.0025$
	Descriptive	TTX (n = 7 pMNs): 33.14 ± 2.9<br>TTX+NBQX (n = 7 pMNs): 13.75 ± 1.8<br>TTX+NBQX+AP5 (n = 7 pMNs): 3.6 ± 0.4				
**2i**<br>SCI-7dpi<br>Frequency	RM<br>One-way<br>ANOVA	$F_{(1.52,\ 9.13)} = 142.2$,<br>$P < 0.0001$	Tukey's test	TTX<br>TTX+NBQX	****	$P_{adj} < 0.0001$
				TTX<br>TTX+NBQX+AP5	****	$P_{adj} < 0.0001$
				TTX+NBQX<br>TTX+NBQX+AP5	**	$P_{adj} = 0.0016$
	Descriptive	TTX (n = 7 pMNs): 16.74 ± 1.19<br>TTX+NBQX (n = 7 pMNs): 6 ± 0.95<br>TTX+NBQX+AP5 (n = 7 pMNs): 1.95 ± 0.45				
**2j**<br>Control<br>Amplitude	RM<br>One-way<br>ANOVA	$F_{(1.297,\ 6.48)} = 66.22$<br>$P < 0.0001$	Tukey's test	TTX<br>TTX+NBQX	**	$P_{adj} = 0.0043$
				TTX<br>TTX+NBQX+AP5	***	$P_{adj} = 0.0003$
				TTX+NBQX<br>TTX+NBQX+AP5	**	$P_{adj} = 0.0018$
	Descriptive	TTX (n = 6 pMNs): 12.64 ± 0.8<br>TTX+NBQX (n = 6 pMNs): 6.41 ± 0.33<br>TTX+NBQX+AP5 (n = 6 pMNs): 3.11 ± 0.37				
**2j**<br>SCI-3dpi<br>Amplitude	RM<br>One-way<br>ANOVA	$F_{(1.211,\ 7.268)} = 40.39$<br>$P = 0.0002$	Tukey's test	TTX<br>TTX+NBQX	**	$P_{adj} = 0.0051$
				TTX<br>TTX+NBQX+AP5	***	$P_{adj} = 0.0009$
				TTX+NBQX<br>TTX+NBQX+AP5	**	$P_{adj} = 0.0014$
	Descriptive	TTX (n = 7 pMNs): 15.93 ± 1.76<br>TTX+NBQX (n = 7 pMNs): 8.33 ± 0.59<br>TTX+NBQX+AP5 (n = 7 pMNs): 4.47 ± 0.43				
**2j**<br>SCI-7dpi<br>Amplitude	RM<br>One-way<br>ANOVA	$F_{(1.012,\ 4.048)} = 22.0$<br>$P = 0.009$	Tukey's test	TTX<br>TTX+NBQX	**	$P_{adj} = 0.0063$
				TTX<br>TTX+NBQX+AP5	*	$P_{adj} = 0.0231$
				TTX+NBQX<br>TTX+NBQX+AP5	ns	$P_{adj} = 0.1686$
	Descriptive	TTX (n = 5 pMNs): 12.48 ± 1.59<br>TTX+NBQX (n = 5 pMNs): 5.69 ± 0.63<br>TTX+NBQX+AP5 (n = 5 pMNs): 3.47 ± 0.48				
**3b**	One-way<br>ANOVA	$F_{(2,\ 73)} = 16.03$<br>$P < 0.0001$	Dunnett's test	Control<br>SCI-3dpi	****	$P_{adj} < 0.0001$
				Control<br>SCI-7dpi	ns	$P_{adj} = 0.4202$
	Descriptive	Control (n = 27 pMNs): 0.696 ± 0.03<br>SCI-3dpi (n = 30 pMNs): 0.48 ± 0.03<br>SCI-7dpi (n = 19 pMNs): 0.76 ± 0.03				
**3c**<br>RMP	One-way<br>ANOVA	$F_{(2,\ 92)} = 0.833$			ns	*P = 0.4377*
	Descriptive	Control (n = 38 neurons): -64.2 ± 0.6<br>SCI-3dpi (n = 35 neurons): -63.76 ± 0.8<br>SCI-7dpi (n = 22 neurons): -65.47 ± 1.2				
**3c**<br>Rinput	One-way<br>ANOVA	$F_{(2,\ 92)} = 18.49$,<br>$P < 0.0001$	Tukey's test	Control<br>SCI-3dpi	****	$P_{adj} < 0.0001$
				Control<br>SCI-7dpi	ns	$P_{adj} = 0.5731$
				SCI-3dpi<br>SCI-7dpi	****	$P_{adj} < 0.0001$
	Descriptive	Control (n = 38 neurons): 0.04 ± 0.002<br>SCI-3dpi (n = 35 neurons): 0.06 ± 0.004<br>SCI-7dpi (n = 22 neurons): 0.036 ± 0.002				
**3c**<br>Rheobase	One-way<br>ANOVA	$F_{(2,\ 91)} = 9.929$,<br>$P = 0.0001$	Tukey's test	Control<br>SCI-3dpi	***	$P_{adj} = 0.0007$

				Control<br>SCI-7dpi	ns	$P_{adj} = 0.9126$
				SCI-3dpi<br>SCI-7dpi	***	$P_{adj} = 0.0010$
	Descriptive	Control (*n* = 37 neurons): 1603 ± 73.7<br>SCI-3dpi (*n* = 35 neurons): 1202 ± 80.5<br>SCI-7dpi (*n* = 22 neurons): 1651 ± 78				
**3e**<br>Frequency	Unpaired<br>*t*-test	t = 4.397, df = 15 (Two-tailed)		Control<br>SCI-3dpi	***	*P = 0.0005*
	Descriptive	Control (*n* = 7 neurons): 0.07 ± 0.01<br>SCI-3dpi (*n* = 10 neurons): 0.16 ± 0.01				
**3e**<br>Amplitude	Unpaired<br>*t*-test	t = 6.053, df = 129 (Two-tailed)		Control<br>SCI-3dpi	****	$P < 0.0001$
	Descriptive	Control (*n* = 28 events): 21.24 ± 1.31<br>SCI-3dpi (*n* = 103 events): 33.12 ± 0.95				
**3f**	Unpaired<br>*t*-test	t = 8.91, df = 128 (Two-tailed)		Control<br>SCI-3dpi	****	$P < 0.0001$
	Descriptive	Control (*n* = 70 events): 27.44 ± 1.16<br>SCI-3dpi (*n* = 60 events): 44.47 ± 1.55				
**3g**<br>Recovery time	Unpaired<br>*t*-test	t = 5.68, df = 15 (Two-tailed)		Control<br>SCI-3dpi	****	$P < 0.0001$
	Descriptive	Control (*n* = 7 events): 17.09 ± 1.1<br>SCI-3dpi (*n* = 10 events): 27.98 ± 1.39				
**3g**<br>Amplitude	Unpaired<br>*t*-test	t = 6.4, df = 15 (Two-tailed)		Control<br>SCI-3dpi	****	$P < 0.0001$
	Descriptive	Control (*n* = 7 events): 37.31 ± 1.81<br>SCI-3dpi (*n* = 10 events): 53.71 ± 1.72				
**3h**	Unpaired<br>*t*-test	t = 6.682, df = 12 (Two-tailed)		Control<br>SCI-3dpi	****	$P < 0.0001$
	Descriptive	Control (*n* = 7 zebrafish): 1.185 ± 0.03<br>SCI-3dpi (*n* = 7 zebrafish): 2.018 ± 0.11				
**3h**	Linear regression	**<u>Control</u>**<br>$R^2 = 0.057$<br>Sy.x = 13.97<br>Equation: Y= 0.09974*X + 26.18<br><br>**<u>SCI-3dpi</u>**<br>$R^2 = 0.000085$<br>Sy.x = 25.16<br>Equation: Y= -0.00558*X + 88.74				
**3i**	One-way ANOVA	$F_{(3, 27)} = 4.904$<br>$P = 0.0076$	Dunnett's test	Control<br>SCI-1dpi	**	$P_{adj} = 0.0076$
				Control<br>SCI-3dpi	ns	$P_{adj} = 0.1118$
				Control<br>SCI-7dpi	**	$P_{adj} = 0.0047$
	Descriptive	Control (*n* = 6 zebrafish): 0.39 ± 0.03<br>SCI-1dpi (*n* = 9 zebrafish): 0.61 ± 0.04<br>SCI-3dpi (*n* = 8 zebrafish): 0.53 ± 0.04<br>SCI-7dpi (*n* = 8 zebrafish): 0.63 ± 0.05				
**3j**<br>Intensity	Unpaired<br>*t*-test	t = 13.34, df = 17 (Two-tailed)		Control<br>SCI-3dpi	****	$P <0.0001$
	Descriptive	Control (*n* = 10 zebrafish): 0.415 ± 0.01<br>SCI-3dpi (*n* = 9 zebrafish): 0.729 ± 0.01				
**3j**<br>Puncta	Unpaired<br>*t*-test	t = 19.31, df = 17 (Two-tailed)		Control<br>SCI-3dpi	****	$P <0.0001$
	Descriptive	Control (*n* = 10 zebrafish): 38.18 ± 1.44<br>SCI-3dpi (*n* = 9 zebrafish): 112.9 ± 3.76				
**4c**<br>Intensity	One-way ANOVA	$F_{(3, 37)} = 23.89$<br>$P < 0.0001$	Dunnett's test	Control<br>SCI-1dpi	*	$P_{adj} = 0.0176$
				Control<br>SCI-3dpi	**	$P_{adj} = 0.0091$
				Control<br>SCI-7dpi	***	$P_{adj} = 0.0002$
	Descriptive	Control (*n* = 11 zebrafish): 0.29 ± 0.007<br>SCI-1dpi (*n* = 9 zebrafish): 0.4 ± 0.03<br>SCI-3dpi (*n* = 11 zebrafish): 0.4 ± 0.03<br>SCI-7dpi (*n* = 10 zebrafish): 0.14 ± 0.01				
**4c**<br>Puncta	One-way ANOVA	$F_{(3, 37)} = 52.34$,<br>$P < 0.0001$	Dunnett's test	Control<br>SCI-1dpi	ns	$P_{adj} = 0.1193$

				Control<br>SCI-3dpi	****	P_{adj} <0.0001
				Control<br>SCI-7dpi	***	P_{adj} = 0.0008
	Descriptive	Control (*n* = 11 zebrafish): 156.8 ± 3.12<br>SCI-1dpi (*n* = 9 zebrafish): 176.2 ± 6.73<br>SCI-3dpi (*n* = 11 zebrafish): 231.9 ± 9.7<br>SCI-7dpi (*n* = 10 zebrafish): 119.9 ± 4.21				
**4f**	One-way ANOVA	$F_{(2, 37)}$ = 15.86,<br>*P* < 0.0001	Tukey's test	Control<br>SCI-3dpi	****	P_{adj} <0.0001
				Control<br>SCI-7dpi	ns	P_{adj} = 0.0526
				SCI-3dpi<br>SCI-7dpi	*	P_{adj} = 0.0178
	Descriptive	Control (*n* = 18 pairs): 4.72 ± 0.27<br>SCI-3dpi (*n* = 11 pairs): 8.26 ± 0.6<br>SCI-7dpi (*n* = 11 pairs): 6.24 ± 0.58				
**4j**<br>Dye coupled cells	One-way ANOVA	$F_{(2, 32)}$ = 14.03,<br>*P* < 0.0001	Dunnett's test	Control<br>SCI-3dpi	***	P_{adj} = 0.0001
				Control<br>SCI-7dpi	ns	P_{adj} = 0.8345
	Descriptive	Control (*n* = 13 zebrafish): 20.1 ± 1.2<br>SCI-3dpi (*n* = 16 zebrafish): 31.3 ± 2<br>SCI-7dpi (*n* = 6 zebrafish): 18.5 ± 1.6				
**4j**<br>Soma size	One-way ANOVA	$F_{(2, 340)}$ = 1.44			ns	*P* = 0.2379
	Descriptive	Control (*n* = 83 neurons): 69.5 ± 7.89<br>SCI-3dpi (*n* = 167 neurons): 56.5 ± 4.44<br>SCI-7dpi (*n* = 63 neurons): 68.2 ± 9.43				
**4m**<br>Control	Paired *t*-test	t = 0.941, df = 2 (Two-tailed)		From pMN<br>To pMN	ns	*P* = 0.4461
	Descriptive	From pMN (*n* = 3 pairs): 2.77 ± 0.7<br>To pMN (*n* = 3 pairs): 2.53 ± 0.49<br>To pMN – From pMN (*n* = 3 pairs): -0.2 ± 0.24				
**4m**<br>SCI-3dpi	Paired *t*-test	t = 3.353, df = 4 (Two-tailed)		From pMN<br>To pMN	*	*P* = 0.0285
	Descriptive	From pMN (*n* = 5 pairs): 5.073 ± 0.8<br>To pMN (*n* = 5 pairs): 6.212 ± 0.69<br>To pMN – From pMN (*n* = 5 pairs): 1.13 ± 0.33				
**5b**<br>intensity	One-way ANOVA	$F_{(2, 14)}$ = 29.66<br>*P* < 0.0001	Dunnett's test	saline<br>NMDA+AMPA	ns	P_{adj} = 0.9379
				saline<br>LY379268	****	P_{adj} <0.0001
	Descriptive	saline (*n* = 7 zebrafish): 0.45 ± 0.03<br>NMDA+AMPA (*n* = 3 zebrafish): 0.43 ± 0.04<br>LY379268 (*n* = 7 zebrafish): 0.76 ± 0.03				
**5b**<br>puncta	One-way ANOVA	$F_{(2, 14)}$ = 21.09<br>*P* < 0.0001	Dunnett's test	saline<br>NMDA+AMPA	ns	P_{adj} = 0.9882
				saline<br>LY379268	****	P_{adj} <0.0001
	Descriptive	saline (*n* = 7 zebrafish): 152.8 ± 3.5<br>NMDA+AMPA (*n* = 3 zebrafish): 151.7 ± 4.03<br>LY379268 (*n* = 7 zebrafish): 190.1 ± 5.5				
**5c**<br>coupling coefficient	One-way ANOVA	$F_{(2, 49)}$ = 6.257,<br>*P* = 0.0038	Dunnett's test	saline<br>NMDA+AMPA	ns	P_{adj} > 0.9999
				saline<br>LY379268	**	P_{adj} = 0.0021
	Descriptive	saline (*n* = 36 pMNs): 4.72 ± 0.2<br>NMDA+AMPA (*n* = 4 pMNs): 4.71 ± 0.59<br>LY379268 (*n* = 12 pMNs): 6.8 ± 0.6				
**5e**<br>intensity	One-way ANOVA	$F_{(2, 15)}$ = 18.64<br>*P* < 0.0001	Dunnett's test	saline<br>LY379268	**	P_{adj} = 0.0052
				saline<br>LY341495	ns	P_{adj} = 0.0955
	Descriptive	saline (*n* = 5 zebrafish): 0.6 ± 0.03<br>LY379268 (*n* = 6 zebrafish): 0.78 ± 0.029<br>LY341495 (*n* = 7 zebrafish): 0.5 ± 0.03				
**5e**<br>puncta	One-way ANOVA	$F_{(2, 15)}$ = 30.27<br>*P* < 0.0001	Dunnett's test	saline<br>LY379268	**	P_{adj} = 0.0015

				saline<br>LY341495	*	P_{adj} = 0.0147
	Descriptive	saline (*n* = 5 zebrafish): 227.4 ± 12.89<br>LY379268 (*n* = 6 zebrafish): 278.6 ± 5.21<br>LY341495 (*n* = 7 zebrafish): 191.4 ± 6.82				
**5f**<br>coupling coefficient	One-way ANOVA	$F_{(2, 47)}$ = 16.38<br>*P* < 0.0001	Dunnett's test	saline<br>LY379268	**	P_{adj} = 0.0092
				saline<br>LY341495	**	P_{adj} = 0.0030
	Descriptive	saline (*n* = 22 pMNs): 8.26 ± 0.4<br>NMDA+AMPA (*n* = 12 pMNs): 10.61 ± 0.56<br>LY379268 (*n* = 16 pMNs): 5.83 ± 0.56				
**6b**	Unpaired *t*-test	t = 2.645, df = 11 (Two-tailed)		Control<br>SCI-3dpi	*	*P* = 0.0228
	Descriptive	Control (*n* = 7 zebrafish): 74.19 ± 1.2<br>SCI-3dpi (*n* = 6 zebrafish): 78.97 ± 1.2				
**6c**<br>Neurons	Unpaired *t*-test	t = 6.85, df = 11 (Two-tailed)		Control<br>SCI-3dpi	****	*P* <0.0001
	Descriptive	Control (*n* = 7 zebrafish): 16.57 ± 1.3<br>SCI-3dpi (*n* = 6 zebrafish): 27.67 ± 0.8				
**6c**<br>Soma sizes	Unpaired *t*-test	t = 0.223, df = 101 (Two-tailed)		Control CR+<br>SCI-3dpi CR+	ns	*P* = 0.8235
	Descriptive	Control CR+ (*n* = 51 neurons): 131.9 ± 10.3<br>SCI-3dpi CR+ (*n* = 52 neurons): 128.3 ± 11.9				
	Unpaired *t*-test	t = 2.748, df = 287 (Two-tailed)		Control CR-<br>SCI-3dpi CR-	**	*P* = 0.0064
	Descriptive	Control CR- (*n* = 129 neurons): 37.8 ± 1.1<br>SCI-3dpi CR- (*n* = 160 neurons): 42.7 ± 1.3				
**6d**	Unpaired *t*-test	t = 3.247, df = 7 (Two-tailed)		Saline<br>CBX	*	*P* = 0.0141
	Descriptive	Saline (*n* = 5 zebrafish): 0.499 ± 0.06<br>CBX (*n* = 4 zebrafish): 0.76 ± 0.04				
**6e**	Unpaired *t*-test	t = 3.315, df = 11 (Two-tailed)		w/o CBX<br>CBX	**	*P* = 0.0069
	Descriptive	w/o CBX (*n* = 8 zebrafish): 0.537 ± 0.04<br>CBX (*n* = 5 zebrafish): 0.758 ± 0.03				
**7c**<br>Ratio	One-way ANOVA	$F_{(2, 14)}$ = 19.35<br>*P* < 0.0001	Dunnett's test	Control<br>SCI-3dpi	****	P_{adj} <0.0001
				Control<br>SCI-7dpi	ns	P_{adj} = 0.8512
	Descriptive	Control (*n* = 9 zebrafish): 24.33 ± 2.1<br>SCI-3dpi (*n* = 5 zebrafish): 51.6 ± 4.1<br>SCI-7dpi (*n* = 3 zebrafish): 27 ± 6.4				
**7c**<br>Soma size	One-way ANOVA	$F_{(2, 383)}$ = 9.761<br>*P* < 0.0001	Dunnett's test	Control<br>SCI-3dpi	*	P_{adj} = 0.0424
				Control<br>SCI-7dpi	ns	P_{adj} = 0.1171
				SCI-3dpi<br>SCI-7dpi	****	P_{adj} <0.0001
	Descriptive	Control (*n* = 115 neurons): 38.16 ± 1.8<br>SCI-3dpi (*n* = 194 neurons): 44.4 ± 1.79<br>SCI-7dpi (*n* = 77 neurons): 31.74 ± 1.7				
**7d**	Unpaired *t*-test	t = 4.137, df = 8 (Two-tailed)		Saline<br>CBX	**	*P* = 0.0033
	Descriptive	Saline (*n* = 6 zebrafish): 75.67 ± 8.8<br>CBX (*n* = 4 zebrafish): 207.8 ± 37.8				
**7f**<br>Neurons	Unpaired *t*-test	t = 7.06, df = 8 (Two-tailed)		Saline<br>CBX	***	*P* = 0.0001
	Descriptive	Saline (*n* = 6 zebrafish): 0.5 ± 0.22<br>CBX (*n* = 4 zebrafish): 7.75 ± 1.25				
**7f**<br>Soma sizes	Unpaired *t*-test	t = 0.67, df = 32 (Two-tailed)		Saline<br>CBX	ns	*P* = 0.5063
	Descriptive	Saline (*n* = 3 cells): 33.6 ± 4.6<br>CBX (*n* = 31 cells): 38.8 ± 2.3				
**7h**	Unpaired *t*-test	t = 2.093, df = 29 (Two-tailed)		Control – 7dpi<br>CBX– 7dpi	*	*P* = 0.0452
	Descriptive	Saline – 7dpi (*n* = 17 zebrafish): 0.08 ± 0.02<br>CBX – 7dpi (*n* = 14 zebrafish): 0.019 ± 0.01				
	Unpaired *t*-test	t = 3.817, df = 29 (Two-tailed)		Control – 14dpi<br>CBX– 14dpi	***	*P* = 0.0007

	Descriptive	Saline – 14dpi (*n* = 19 zebrafish): 0.71 ± 0.03<br>CBX – 14dpi (*n* = 12 zebrafish): 0.51 ± 0.03				
	Unpaired *t*-test	t = 0.47, df = 19 (Two-tailed)		Control – 21dpi<br>CBX– 21dpi	ns	*P* = 0.6427
	Descriptive	Saline – 21dpi (*n* = 10 zebrafish): 0.83 ± 0.04<br>CBX – 21dpi (*n* = 11 zebrafish): 0.8 ± 0.03				
**7j**	Unpaired *t*-test	t = 2.139, df = 13 (Two-tailed)		Saline<br>CBX	ns	*P* = 0.0502
	Descriptive	Saline – 21dpi (*n* = 7 zebrafish): 4.28 ± 0.8<br>CBX – 21dpi (*n* = 8 zebrafish): 2 ± 0.65				
**Supplementary figures**						
**Supplementary 1d** Branches	Unpaired *t*-test	t = 9.777, df = 32 (Two-tailed)		pMNs<br>sMNs	****	*P* <0.0001
	Descriptive	pMNs (*n* = 12 neurons): 25.8 ± 1.8<br>sMNs (*n* = 22 neurons): 9.04 ± 0.8				
**Supplementary 1d** Length	Unpaired *t*-test	t = 12.41, df = 32 (Two-tailed)		pMNs<br>sMNs	****	*P* <0.0001
	Descriptive	pMNs (*n* = 12 neurons): 1124 ± 82.6<br>sMNs (*n* = 22 neurons): 255.8 ± 26				
**Supplementary 1d** Area	Unpaired *t*-test	t = 12.36, df = 32 (Two-tailed)		pMNs<br>sMNs	****	*P* <0.0001
	Descriptive	pMNs (*n* = 12 neurons): 7021 ± 486<br>sMNs (*n* = 22 neurons): 1548 ± 194				
**Supplementary 2a** Number	One-way ANOVA	$F_{(3, 26)}$ = 0.0166			ns	*P* = 0.997
	Descriptive	Control (*n* = 12 zebrafish): 48.17 ± 1<br>SCI-1dpi (*n* = 4 zebrafish): 47.75 ± 1.5<br>SCI-3dpi (*n* = 9 zebrafish): 48 ± 1.05<br>SCI-7dpi (*n* = 5 zebrafish): 48.2 ± 2.2				
**Supplementary 2b** Size	One-way ANOVA	$F_{(3, 632)}$ = 1.223			ns	*P* = 0.3006
	Descriptive	Control (*n* = 214 sMNs): 63.62 ± 1.6<br>SCI-1dpi (*n* = 162 sMNs): 65.84 ± 2<br>SCI-3dpi (*n* = 178 sMNs): 65.93 ± 1.9<br>SCI-7dpi (*n* = 82 sMNs): 69.73 ± 2.5				
**Supplementary 5b** Peak	One-way ANOVA	$F_{(2, 92)}$ = 1.238			ns	*P* = 0.2947
	Descriptive	Control (*n* = 38 neurons): 35.2 ± 2.6<br>SCI-3dpi (*n* = 35 neurons): 31.4 ± 2.3<br>SCI-7dpi (*n* = 22 neurons): 29.4 ± 2.6				
**Supplementary 5b** Threshold	Descriptive	$F_{(2, 92)}$ = 3.493, *P* = 0.0345	Tukey's test	Control<br>SCI-3dpi	ns	P_{adj} = 0.5380
				Control<br>SCI-7dpi	*	P_{adj} = 0.0259
				SCI-3dpi<br>SCI-7dpi	ns	P_{adj} = 0.2165
	Descriptive	Control (*n* = 38 neurons): 8.8 ± 2.9<br>SCI-3dpi (*n* = 35 neurons): 4.4 ± 2.6<br>SCI-7dpi (*n* = 22 neurons): -3.5 ± 4				
**Supplementary 5b** Amplitude	One-way ANOVA	$F_{(2, 92)}$ = 5.161, *P* = 0.0075	Tukey's test	Control<br>SCI-3dpi	ns	P_{adj} > 0.9999
				Control<br>SCI-7dpi	*	P_{adj} = 0.0123
				SCI-3dpi<br>SCI-7dpi	*	P_{adj} = 0.0138
	Descriptive	Control (*n* = 38 neurons): 26.9 ± 1<br>SCI-3dpi (*n* = 35 neurons): 26.9 ± 1.2<br>SCI-7dpi (*n* = 22 neurons): 32.5 ± 1.7				
**Supplementary 5b** Half width	One-way ANOVA	$F_{(2, 92)}$ = 1.113			ns	*P* = 0.3330
	Descriptive	Control (*n* = 38 neurons): 0.31 ± 0.01<br>SCI-3dpi (*n* = 35 neurons): 0.32 ± 0.01<br>SCI-7dpi (*n* = 22 neurons): 0.33 ± 0.01				
**Supplementary 5b** AHP amplitude	One-way ANOVA	$F_{(2, 92)}$ = 3.105, *P* = 0.0496	Tukey's test	Control<br>SCI-3dpi	ns	P_{adj} = 0.4900
				Control<br>SCI-7dpi	*	P_{adj} = 0.0384
				SCI-3dpi<br>SCI-7dpi	ns	P_{adj} = 0.3117

	Descriptive	Control (*n* = 38 neurons): 8.75 ± 0.4<br>SCI-3dpi (*n* = 35 neurons): 9.5 ± 0.5<br>SCI-7dpi (*n* = 22 neurons): 10.6 ± 0.4				
**Supplementary 5b**<br>Number of AP	One-way ANOVA	$F_{(2, 92)}$ = 4.111,<br>*P* = 0.0195	Tukey's test	Control<br>SCI-3dpi	*	P_{adj} = 0.0162
				Control<br>SCI-7dpi	ns	P_{adj} = 0.2228
				SCI-3dpi<br>SCI-7dpi	ns	P_{adj} = 0.7148
	Descriptive	Control (*n* = 38 neurons): 8.2 ± 1.3<br>SCI-3dpi (*n* = 35 neurons): 18.5 ± 3.4<br>SCI-7dpi (*n* = 22 neurons): 15.2 ± 3.4				
**Supplementary 6b**	Unpaired *t*-test	t = 3.723, df = 10 (Two-tailed)		Control<br>SCI-3dpi	**	*P* = 0.004
	Descriptive	Control (*n* = 6 zebrafish): 64.79 ± 2.03<br>SCI-3dpi (*n* = 6 zebrafish): 88.82 ± 6.12				
**Supplementary 6c**<br>Intensity	Unpaired *t*-test	t = 10.09, df = 288 (Two-tailed)		Control<br>SCI-3dpi	****	*P* <0.0001
	Descriptive	Control (*n* = 150 neurons): 62.86 ± 0.91<br>SCI-3dpi (*n* = 140 neurons): 90.37 ± 2.64				
**Supplementary 6c**<br>Intensity vs size	Simple linear regression	**<u>Control</u>**<br>Equation: *Y = 0.06166*X + 60.63*<br>R^2: 0.008904<br>**<u>SCI-3dpi</u>**<br>Equation: *Y = -0,1102*X + 94,63*<br>R^2: 0.004098				
**Supplementary 7b**	One-way ANOVA	$F_{(3, 14)}$ = 1.001			ns	*P* = 0.2413
	Descriptive	Control (*n* = 5 zebrafish): 0.592 ± 0.03<br>SCI-1dpi (*n* = 3 zebrafish): 0.555 ± 0.01<br>SCI-3dpi (*n* = 5 zebrafish): 0.618 ± 0.03<br>SCI-7dpi (*n* = 5 zebrafish): 0.621 ± 0.01				
**Supplementary 8b**<br>Number of neurons	Unpaired *t*-test	t = 4.729, df = 12 (Two-tailed)		Control<br>SCI-3dpi	***	*P* = 0.0005
	Descriptive	Control (*n* = 8 zebrafish): 82.7 ± 3.1<br>SCI-3dpi (*n* = 6 zebrafish): 124.2 ± 9.3				
**Supplementary 8b**<br>Soma size	Unpaired *t*-test	t = 0.75, df = 1272 (Two-tailed)		Control<br>SCI-3dpi	ns	*P* = 0.4534
	Descriptive	Control (*n* = 502 neurons): 59 ± 2.4<br>SCI-3dpi (*n* = 772 neurons): 61.2 ± 1.8				
**Supplementary 8c**	Unpaired *t*-test	t = 0.1886, df = 12 (Two-tailed)		Control<br>SCI-3dpi	ns	*P* = 0.8536
	Descriptive	Control (*n* = 8 zebrafish): 0.37 ± 0.01<br>SCI-3dpi (*n* = 6 zebrafish): 0.36 ± 0.01				
**Supplementary 8d**	Linear regression	**<u>Control:</u>**<br>R^2 = 0.3376<br>Sy.x = 0.1791<br>Equation: *Y= 0.002144*X + 0.256*<br>**<u>SCI-3dpi:</u>**<br>R^2 = 0.1493<br>Sy.x = 0.1845<br>Equation: *Y= 0.001534*X + 0.254*				
**Supplementary 8f**<br>Number of sMNs	Unpaired *t*-test	t = 2.902, df = 8 (Two-tailed)		Control<br>SCI-3dpi	*	*P* = 0.0198
	Descriptive	Control (*n* = 6 zebrafish): 41.12 ± 0.95<br>SCI-3dpi (*n* = 4 zebrafish): 45.6 ± 1.23				
**Supplementary 8f**<br>Intensity	Unpaired *t*-test	t = 0.012, df = 8 (Two-tailed)		Control<br>SCI-3dpi	ns	*P* = 0.9902
	Descriptive	Control (*n* = 6 zebrafish): 0.68 ± 0.008<br>SCI-3dpi (*n* = 4 zebrafish): 0.68 ± 0.009				
**Supplementary 8f**<br>Intensity / size	Simple linear regression	**<u>Control</u>**<br>Equation: *Y = 0.00031*X + 0.628*<br>R^2: 0.0055<br>**<u>SCI-3dpi</u>**<br>Equation: *Y = 0,00039*X + 0.63*<br>R^2: 0.0049				
**Supplementary 8g**	Unpaired *t*-test	t = 0.9929, df = 8 (Two-tailed)		Control<br>SCI-3dpi	ns	*P* = 0.3498

Number of sMNs	Intermediate				
	Descriptive	Control (*n* = 6 zebrafish): 9.84 ± 0.59<br>SCI-3dpi (*n* = 4 zebrafish): 11.11 ± 1.23			
	Unpaired<br>*t*-test<br>Fast	t = 2.761, df = 8 (Two-tailed)	Control<br>SCI-3dpi	*	*P* = 0.024
	Descriptive	Control (*n* = 6 zebrafish): 88.59 ± 0.766<br>SCI-3dpi (*n* = 4 zebrafish): 92.5 ± 1.32			
**Supplementary 8g**<br>Intensity	Unpaired<br>*t*-test<br>Intermediate	t = 0.2758, df = 8 (Two-tailed)	Control<br>SCI-3dpi	ns	*P* = 0.7897
	Descriptive	Control (*n* = 6 zebrafish): 0.62 ± 0.02<br>SCI-3dpi (*n* = 4 zebrafish): 0.64 ± 0.03			
	Unpaired<br>*t*-test<br>Fast	t = 0.2938, df = 8 (Two-tailed)	Control<br>SCI-3dpi	ns	*P* = 0.7764
	Descriptive	Control (*n* = 6 zebrafish): 0.67 ± 0.02<br>SCI-3dpi (*n* = 4 zebrafish): 0.66 ± 0.02			
**Supplementary 9b**	Unpaired<br>*t*-test	t = 12.95, df = 137 (Two-tailed)	pMNs<br>sMNs	****	*P* <0.0001
	Descriptive	pMNs (*n* = 32 neurons): 1523 ± 53.6<br>sMNs (*n* = 107 neurons): 684 ± 31.5			
**Supplementary 9e**	Unpaired<br>*t*-test	t = 10.47, df = 177 (Two-tailed)	pMNs<br>HuC/D	****	*P* <0.0001
	Descriptive	pMNs (*n* = 20 neurons): 1625 ± 87.9<br>HuC/D (*n* = 159 neurons): 576 ± 33.7			
**Supplementary 10b**	Linear regression	R^2 = 0.0444<br>Sy.x = 18.85<br>Equation: Y= -2.434*X + 56.32			
**Supplementary 10c**	Paired<br>*t*-test	t = 0.849, df = 17 (Two-tailed)		ns	*P* = 0.4073
	Descriptive	Rostral to caudal (*n* = 9 pairs): 4.95 ± 0.4<br>Caudal to rostral (*n* = 9 pairs): 4.48 ± 0.3			
**Supplementary 11b**	Unpaired<br>*t*-test	t = 5.639, df = 12 (Two-tailed)	Control<br>SCI-3dpi	***	*P* = 0.0001
	Descriptive	Control (*n* = 8 zebrafish): 4.093 ± 0.55<br>SCI-3dpi (*n* = 6 zebrafish): 10.36 ± 1.06			
**Supplementary 11d**	Descriptive	Distance (*n* = 222 dye-coupled neurons): 5.32 ± 0.41			
**Supplementary 12a**	Paired<br>*t*-test	t = 8, df = 5 (Two-tailed)	Control<br>CBX	***	*P* = 0.0005
	Descriptive	Control: 5.6 ± 0.6<br>CBX: 0.5 ± 0.2			
**Supplementary 12b**	Unpaired<br>*t*-test	t = 3.795, df = 15 (Two-tailed)	Control<br>CBX	**	*P* = 0.0018
	Descriptive	Control (*n* = 13 zebrafish): 20.15 ± 1.2<br>CBX (*n* = 4 zebrafish): 10.25 ± 2.2			
**Supplementary 12c**	Descriptive	200 µM (*n* = 3 zebrafish): 0.000<br>1 mM (*n* = 4 zebrafish): 4.5 ± 1.19<br>5 mM (*n* = 5 zebrafish): 16.8 ± 1.65<br>10 mM (*n* = 5 zebrafish): 32 ± 3.89			

Supplementary Table 2. Antibodies and Streptavidins Used[1]

Antigen	Host	Source	Code	Dilution
Primary				
ChAT	Goat	Millipore	AB144P; RRID: AB_2079751	1:200
HuC/D	Mouse	Molecular Probes	A-21271; RRID: AB_221448	1:500
HuC/D	Rabbit	GeneTex	GTX128365; RRID: AB_2885764	1:400
NeuN	Rabbit	Cell Signaling	#24307; RRID: AB_2651140	1:400
PV	Mouse	Swant	235; RRID: AB_10000343	1:300
CR	Mouse	Swant	6B3; RRID: AB_10000320	1:400-1:800
Cx35/36	Mouse	Millipore	MAB3045; RRID: AB_94632	1:100
Secondary				
Goat IgG-488	Donkey	ThermoFisher	A-11055; RRID: AB_2534102	1:500
Mouse IgG-647	Donkey	ThermoFisher	A-31571; RRID: AB_162542	1:500
Mouse IgG-568	Donkey	ThermoFisher	A-10037; RRID: AB_2534013	1:500
Mouse IgG-488	Donkey	ThermoFisher	A-21202; RRID: AB_141607	1:500
Rabbit IgG-488	Donkey	ThermoFisher	A-21206; RRID: AB_2535792	1:500
Rabbit IgG-647	Donkey	ThermoFisher	A-31573; RRID: AB_2536183	1:500
Rabbit IgG-568	Donkey	ThermoFisher	A-10042; RRID: AB_2534017	1:500
Mouse IgG-Biotinylated	Horse	VectorLaboratories	BA-2000; RRID: AB_2313581	1:200
Rabbit IgG-Biotinylated	Horse	VectorLaboratories	BA-1100; RRID: AB_2336201	1:200
Streptavidin				
Alexa Fluor 488		ThermoFisher	S32354; RRID: AB_2315383	1:500
Alexa Fluor 555		ThermoFisher	S32355; RRID: AB_2571525	1:500
Alexa Fluor 647		ThermoFisher	S32357; RRID: AB_2336066	1:500

[1]ChAT, choline-acetyltransferase; CR, calretinin; Cx35/36, Connexin 35/36; HuC/D, Elav3+4; NeuN, Neuronal protein Fox-3/RBFOX3; PV, parvalbumin.
